# GEO Data Sets Analysis Identifies COX-2 and Its Related Micro RNAs as Biomarkers for Non-Ischemic Heart Failure

**DOI:** 10.3389/fphar.2020.01155

**Published:** 2020-08-05

**Authors:** Youyou Yan, Dandan Song, Xiaoling Zhang, Gang Hui, Junnan Wang

**Affiliations:** ^1^ Department of Cardiology, Second Hospital of Jilin University, Changchun, China; ^2^ Department of Clinical Laboratory, Second Hospital of Jilin University, Changchun, China; ^3^ Key Laboratory of Organ Regeneration and Transplantation of Ministry of Education, Institute of Immunology, The First Hospital, Jilin University, Changchun, China; ^4^ The Department of 31656 Troops of Chinese People's Liberation Army, Leshan, China

**Keywords:** heart failure, micro RNA, COX-2, biomarker, bioinformatics analysis

## Abstract

Heart failure (HF) is a heterogeneous clinical syndrome with a variety of causes, risk factors, and pathology. Clinically, only brain natriuretic peptide (BNP) or its precursor N-terminus proBNP (NTproBNP) has been validated for HF diagnosis, but they are also affected by other conditions, such as female gender, renal disease, and acute coronary syndromes, and false low levels in the setting of obesity or flash pulmonary edema. In addition, there is no one biomarker which could encompass all heart failure phenotypes. Advances in bioinformatics have provided us with large databases that characterize the complex genetic and epigenetic changes associated with human diseases. The use of data mining strategies on public access databases to identify previously unknown disease markers is an innovative approach to identify potential biomarkers or even new therapeutic targets in complex diseases such as heart failure (HF). In this study, we analyzed the genomic and transcription data of HF peripheral blood mononuclear cell (PBMC) samples obtained from the Gene Expression Omnibus data sets using Omicsbean online database (http://www.omicsbean.cn/) and found that the prostaglandin-endoperoxide synthase 2 (PTGS2), also named as cyclooxygenase-2 (COX-2), as well as its related micro RNAs including miR-1297 and miR-4649-3p might be used as potential biomarkers for non-ischemic heart failure. Our result showed that plasma COX-2 and miR-4649-3p were significantly up-regulated, whereas the plasma miR-1297 was significantly decreased, and miR-4649-3p displayed high predictive power for non-ischemic heart failure.

## Introduction

Heart failure (HF) is a heterogeneous clinical syndrome with a variety of risk factors, and pathology and a leading cause of mortality in the world (Linsenmayer et al., 2019). Approximately 50% of patients with HF are expected to die within 5 years of diagnosis (Gathright et al., 2017). Clinically, only brain natriuretic peptide (BNP) or its precursor N-terminus proBNP (NTproBNP) has been validated for HF diagnosis, and they were affected by many other conditions such as advanced age, female gender, renal disease and acute coronary syndromes, and obesity or flash pulmonary edema (Goetze et al., 2005). In addition, the diagnosis of heart failure is most frequently made at the time of presentation of symptoms, while advances in congestive heart failure (CHF) management depend on biomarkers for monitoring disease progression and therapeutic response (Inamdar and Inamdar, 2016). However, there is no biomarker that could encompass all heart failure phenotypes (Senthong et al., 2017). Hence, it is urgent to discover novel and reliable biomarkers for HF.

It was found that some inflammatory markers such as interleukin-33 (IL-33)/ST2 (suppressor of tumorigenicity 2; interleukin 1 receptor-like 1), IL-6, and tumor necrosis factor-alpha (TNF-α), were associated with HF, but they were not specific for HF and were often affected by other multiple comorbidities (Weinberg et al., 2002; Liu et al., 2014). In addition, microRNAs (miRNAs) are small non-coding RNA molecules that can regulate gene expression in many biological processes and stable in blood, those prepotencies make micro RNAs as potential biomarkers (Maegdefessel, 2014). Many studies have investigated the circulating micro RNAs as the biomarkers for cardiovascular diseases including HF (Dickinson et al., 2013; Endo et al., 2013).

Recently, gene expression profiling and the analysis of protein-protein interaction (PPI) networks have been widely used for identifying disease biomarkers and potential drug targets (Sakharkar et al., 2019). Some widely available open access databases such as Gene Expression Omnibus (GEO) provided abundant microarray resources for searching deregulated genes (Sakharkar et al., 2019). Therefore, data mining strategies on public access databases on identifying candidate gene or their related micro RNAs are used to find biomarkers for HF.

In this study, we explored the deregulated genes of non-ischemic heart failure based on Gene Expression Omnibus (GEO) data sets and predicted related micro RNAs using bioinformatics analyses. Furthermore, we tested their levels and evaluated their predictive power for non-ischemic heart failure.

## Methods

### Analysis of Non-Ischemic Heart Failure From GEO Data

Gene Expression Omnibus database (http://www.ncbi.nlm.nih.gov/geo) is an open functional genomics database of high-throughput resource (Barrett et al., 2005). In this study, we downloaded the microarray data of GDS3115 including three non-ischemic heart failure patients and three normal subjects were obtained from the GEO. The microarray data of GDS3115 contained a total of 22214 known genes were then screened with the following criteria: differential expression ratio > 4 (log2FC > 2) or < _4 (log2FC >_2) and a p-value <0.05. The dysregulated gene-related micro RNA prediction and integrating protein-protein-micro RNA interaction were next analyzed by Omicsbean online database (http://www.omicsbean.cn/), which is a multi-omics data analysis tool that integrates biological data and analysis tools and provides a comprehensive set of functional annotation information of genes and proteins for users to extract biological information (Liu et al., 2018).

### Sample Collection

In this study, we enrolled 70 patients that were diagnosed with non-ischemic heart failure at Second Hospital of Jilin University, Changchun, China, From January 2018 to August 2018. Inclusion criteria: Newly diagnosed HF within 12 months in stages B, C, and D according to the American College of Cardiology/American Heart Association 2005 Guidelines (Hunt et al., 2009; Dai et al., 2012), an elevated level (>200 pg. per milliliter) of N-terminal pro-brain natriuretic peptide (NT-proBNP) and a left ventricular ejection fraction (LVEF)** **≤** **35%. Exclusion criteria: advanced HF needed the ongoing support or surgery within 6 months, or HF with prior ST-segment elevation myocardial infarction (STEMI) or non-STEMI in the left anterior descending coronary artery distribution. In addition, 77 matched control subjects without heart failure were used as control. All blood samples (5 ml per patient), collected before those received any treatments, were collected *via* a direct venous puncture and placed into tubes containing sodium citrate, centrifuged at 1,000×***g*** for 5 min and 3,000×***g*** for 10 min, the layer of the supernatant (plasma) was carefully transferred into other tubes and stored at −80**°**C. Written consent was obtained from all subjects, and the study protocol was approved by the ethics committee of Jilin University second hospital.

### Assay for Plasma PTGS2 (COX-2), hsa-miR-4649-3p, and hsa-miR-1297

We test the plasma level of COX-2 using the ELISA kit from Elabscience, following the manufacturer’s instructions. Absorbance was measured at 450 nm (primary wave length).

MiRNAs were extracted from the plasma samples contained 50 pmol/L *Caenorhabditis elegans* miR-39 (cel-miR-39), which was used as an external reference, following the instruction of miRcute miRNA Isolation kit (TRANS GEN, Beijing, China). Each sample was eluted in 100 μl of RNAse-free water. QPCR assay Poly-(A) tailing and reverse transcription were performed with the miScript reverse transcription kit (TRANS GEN, Beijing, China). QRT-PCR was performed to quantify the levels of miRNA using the SYBR Green PCR method (TRANS GEN, Beijing, China). Cel-miR39 was used as a stable exogenous control. Threshold cycle values (Ct values) were determined from amplification curves. Negative controls using nuclease-free water were included with every real-time PCR operation and Ct values >35 viewed as negative. All samples for miRs were run in one assay, and all reactions were run in triplicate. The 2−ΔCt method was used to calculate relative quantitative expression (ΔCt = CtmiRNA−CtmiR39). The micro RNA assay primers used were miR-4649-3p forward: 5′-TCTGAGGCCTGCCTCTCCCA-3′, miR-1297 5′-TTCAAGTAATTCAGGTG-3′ and cel-miR-39 forward: 5′-TCACCGGGUGUAAATCAGCTTG-3′.

### Statistical Analyses

SPSS version 26 (SPSS Inc., USA) was used to perform the statistical analyses. Data are presented as the mean ± SD and median for the general characteristics of the subjects. Differences between control and heart failure group were assessed using Two-tailed t-tests. The correlations of plasma COX-2 and hsa-miR-4649 and hsa-miR-1297 were also assayed using Spearman ranked correlations. Binary logistical regression analysis were also used to evaluate the predictive powers of plasma PTGS2 (COX-2), hsa-miR-4649 and hsa-miR-1297 for HF. Receiver operating characteristic (ROC) analysis containing the ROC curves and overall model quality were used to evaluate the individual predictive accuracy of the candidate biomarkers. As the validation cohort is relatively small, bootstrap analysis of 1000 iterations was used to correct the false positive findings. Values with a p < 0.05 were considered to indicate statistical significance.

## Results

### Baseline Characteristics

70 patients with non-ischemic heart failure and 77 matched control subjects without cardiovascular disease were enrolled. Their clinical characteristics and biochemical parameters are listed in **Table 1**. Age, sex, hypertension, smoking history, drinking history were not different between non-ischemic heart failure and control groups.

**Table 1:** Clinical characteristics and biochemical parameters of the patients with non-ischemic heart failure.

Variable	Control	Non-ischemic heart failure	P value
	n=77	n=70	
Age (y)	60.79 ± 7.56	62.41 ± 7.23	0.16
Male, n (%)	15 (19.48)	11 (15.71)	0.55
Smoking, n (%)	34 (44.16)	21 (30.00)	0.08
Diabetes, n (%)	29 (37.66)	31 (44.29)	0.42
Hypertension, n (%)	39 (50.65)	44 (62.85)	0.19
Drinking (%)	36 (46.75)	35 (50.00)	0.70

### Deferentially Expressed Genes and miRNAs Networks

**Figure 1:**
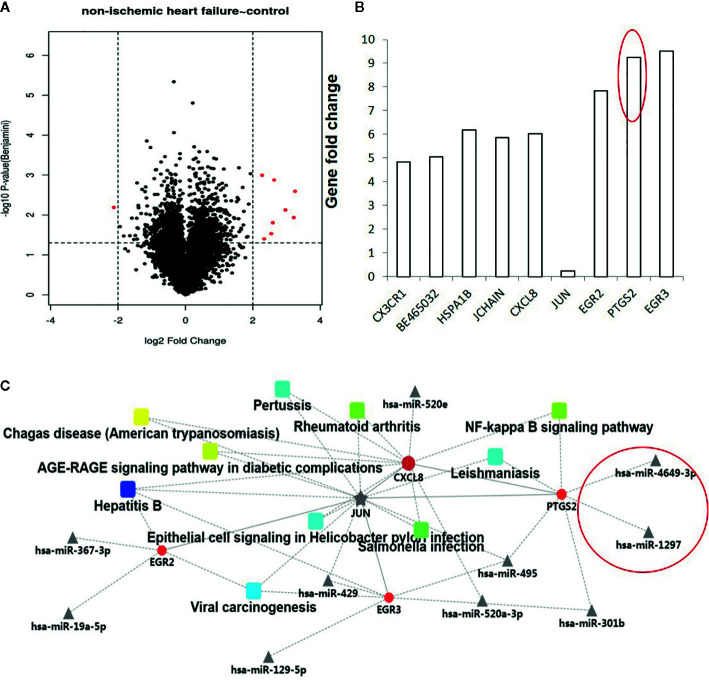
Analysis of dysregulated genes and their micro RNAs between non-ischemic heart failure and control groups. **(A)** The volcano plot graph of genes was constructed according to fold change values and p values. The X axis represents the log2 (fold change) value of differential expression, and the Y axis represents the -log10 (padj) value of differential expression. The vertical lines correspond to 4-fold upregulation and downregulation between non-ischemic heart failure and control groups, and the horizontal line represents a p–value of 0.05. **(B)**. A Histogram of dysregulated genes between non-ischemic heart failure and control groups. **(C)** Construction and analysis of a PPI network.

The gene expression profiles of non-ischemic heart failure and control subjects were down-loaded from GEO data sets and analyzed using the Omicsbean online database (http://www.omicsbean.cn/). Based on the criterions (p < 0.05 and fold change 4), 9 genes were found to be deferentially expressed in non-ischemic heart failure, including 8 up- and 1 downregulated genes (**Figures 1A, B**). Among them, PTGS2 (COX-2) is a secreted protein with high expression levels in patients with non-ischemic heart failure, indicating it might be used as biomarker. As the miRNAs were also investigated as biomarker, the regulation networks of genes and their related miRNAs were constructed based on the gene expression profile. We predicted the dysregulated gene-related micro RNAs and constructed the PPI network to provide the interactions among various proteins and micro RNAs using the Omicsbean online database. Integrative bioinformatics analysis showed that hsa-miR-4649 and hsa-miR-1297 could target COX-2 and were not associated with other dysregulated genes (**Figure 1C**), suggesting they might be also used as the biomarker for no non-ischemic heart failure.

### The Plasma Levels of COX-2 and hsa-miR-4649-3p and hsa-miR-1297 and Their Correlations

**Figure 2:**
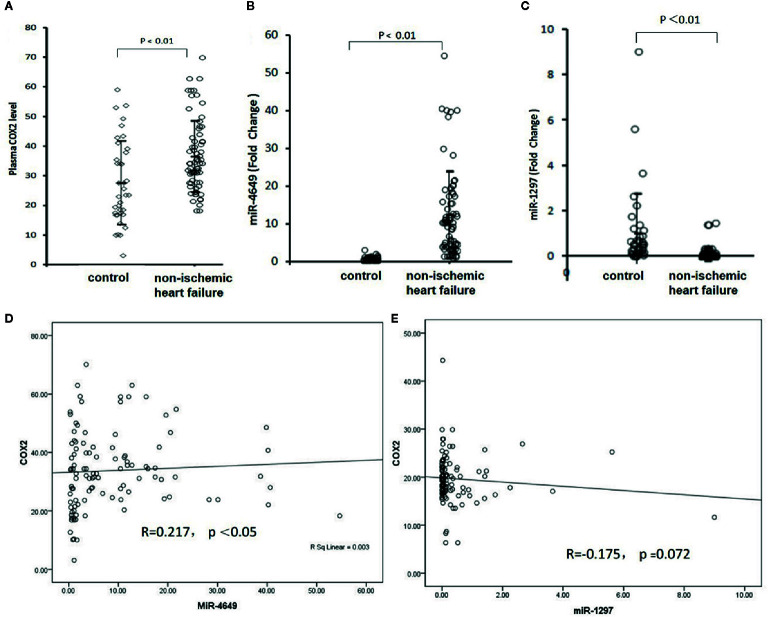
The plasma levels of COX-2, miR-4649-3p, and miR-1297 as well as their correlations. **(A)** The plasma level of COX-2 in patients with non-ischemic heart failure or subjects without cardiovascular disease. **(B)** The plasma level of miR-4649-3p in patients with non-ischemic heart failure or subjects without cardiovascular disease. **(C)** The plasma level of miR-1297 in patients with non-ischemic heart failure or subjects without cardiovascular disease. **(D)** The correlation of COX-2 with miR-4649-3p. **(E)** The correlation of COX-2 with miR-1297.

We next measured the plasma levels of COX-2, hsa-miR-4649-3p, and hsa-miR-1297, and bootstrap analysis of 1000 iterations was used to correct the false positive findings. The results showed that the plasma level of COX-2 in the non-ischemic heart failure group was significantly higher than that in control group (36.67 ± 11.97ng/ml vs 27.84 ± 12.82 ng/ml, 95% CI, −12.88 to −4.78, p = 0.00) (bootstrapped 95% CI, −13.10 to 4.90; p = 0.001) (**Figure 2A**). hsa-miR-4649 was also significantly increased in the plasma of patients with non-ischemic heart failure, compared with control subjects (11.03 ± 10.05 vs 1.00 ± 0.79 fold, 95% CI, −12.43 to −7.63; p = 0.00) (bootstrapped 95% CI, −12.47 to −7.87; p = 0.001) (**Figure 2B**). However, hsa-miR-1297 was significantly decreased in the plasma of patients with non-ischemic heart failure, compared with control subjects (0.13 ± 0.30 vs 1.00 ± 1.33 fold, 95% CI: 0.56,1.18; p =0.00) (bootstrapped 95% CI: 0.56, 1.18; p =0.003) (**Figure 2C**). We further investigated the correlations of COX-2 with hsa-miR-4649 and hsa-miR-1297. The results showed that plasma COX-2 positively correlated with plasma hsa-miR-4649 (R = 0.227, p =0.006) (bootstrapped 95% CI: 0.078, 0.369) (**Figure 2D**). There was a negative correlation of COX-2 with hsa-miR-1297 in non-ischemic heart failure (R = −0.237, p = 0.004) (bootstrapped 95% CI, −0.390 to −0.069) (**Figure 2E**).

### The Predictive Powers of COX-2 With Its Related miRNAs for Non-Ischemic Heart Failure

**Figure 3:**
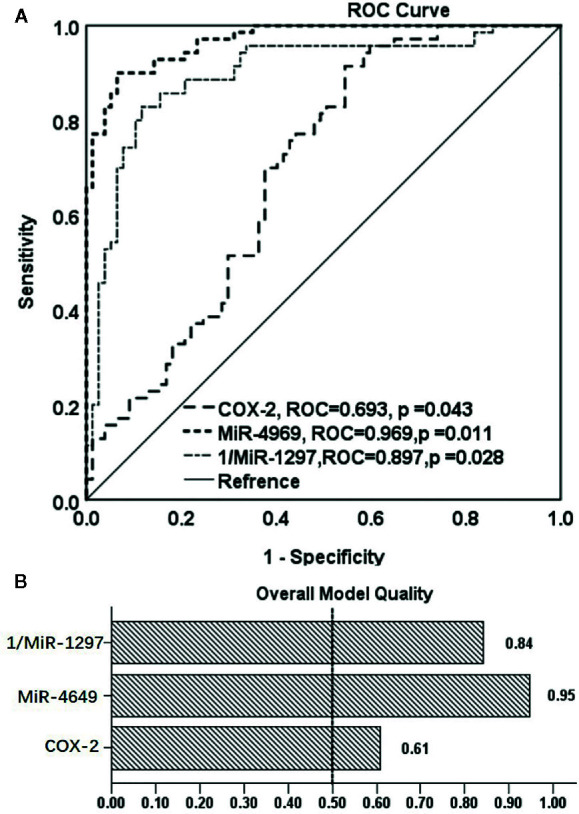
The ROC analysis of the predictive powers of COX-2, miR-4649-3p, and miR-1297 for non-ischemic heart failure. **(A)** ROC curve of COX-2, miR-4649-3p, and 1/miR-12971 for non-ischemic heart failure, **(B)** Overall model quality of COX-2, miR-4649-3p, and 1/miR-12971 for non-ischemic heart failure.

As plasma COX-2 and miR-4649-3p, miR-1297 were significantly dysregulated in patients with non-ischemic heart failure, we evaluated their predictive power by performing ROC analysis. As shown in **Figure 3A**, AUC of COX-2 is 0.693 (95% CI: 0.608, 0.778; p < 0.01), the optimal cutoff value is 31.06 ng/ml with sensitivity, and specificity were 70.0% and 62.3%, respectively. AUC of miR-4649 is 0.969 (95% CI: 0.947, 0.991; p<0.01), and the optimal cutoff value is 2.14 fold change with the sensitivity and specificity are 90.0% and 94.5%, respectively. The AUC of 1/miR-1297 is 0.897 (95% CI: 0.842, 0.951; p<0.01) and the optimal cut-off value is 0.16 fold change with the sensitivity and specificity are 85.7% and 84.4%, respectively. Overall model quality displays the value of the lower bound of the confidence interval of the estimated AUC, and predictive model is considered good when the value is over 0.5. As shown in **Figure 3B**, the overall model quality for COX-2, miR-4649-3p, or miR-1297 is over 0.61, 0.95, 0.84, respectively. Binary logistical regression analysis showed that plasma COX-2 (odds ratio, 1.292; 95% CI, 1.001−1.134; p = 0.047), miR-4649-3p (odds ratio, 3.821; 95% CI, 2.010–7.263; p = 0.000), miR-1297 (odds ratio, 0.031; 95% CI, 0.002–0.514; p = 0.015) were significantly correlated with non-ischemic heart failure (**Table 2**). However, bootstrap analysis of 1000 iterations showed that there was no significant predictive power of plasma COX-2 for non-ischemic heart failure (bootstrapped 95% CI, −0.029 to 0.245; p=0.089) and plasma miR-4649-3p also displayed higher predictive power than miR-1297 ((bootstrapped 95% CI, 0.916–15.783; p=0.002) vs bootstrapped 95% CI: −80.258 to −1.420; p =0.041)).

**Table 2:** Binary logistical regression analysis of COX-2, miR-4649-3p, miR-1297 in patients with non-ischemic heart failure.

Variable	P value	OR	95% CI		Bootstrap	Bootstrap 95% CI	
			Lower	Upper	P-value	Lower	Upper
COX-2	0.047	1.065	1.001	1.134	0.089	−0.029	0.245
MiR-4649-3p	0.000	3.821	2.010	7.263	0.002	0.916	15.783
MiR-1297	0.015	0.031	0.002	0.514	0.041	−80.258	−1.420

Bootstrap significance or 95% CI is two-tailed with 1000 iterations.

## Discussion

Clinically, only BNP or NT-proBNP has been validated for heart failure diagnosis (Goetze et al., 2005), but they are also affected by other conditions and could not encompass all heart failure phenotypes. Recently, data mining strategies on public access databases and integrative bioinformatics analysis have been used to identify potential biomarkers or even new therapeutic targets in complex disease (Sakharkar et al., 2019). In this study, we analyzed GEO Datasets to screen dysregulated genes in whole blood from patients with non-ischemic heart failure for identifying the new biomarkers. We found that COX-2, a secreted protein, was highly expressed in the blood of patients with non-ischemic heart failure. Consistently, many studies showed that COX-2 played an important role in the development of heart failure (Camacho et al., 2011; Liu et al., 2012; Chien et al., 2015; Pang et al., 2016). Normally, the expression of COX-2 is very low and strongly induced by different stimuli, including hypoxia, mechanical stress, and proinflammatory cytokines (Camacho et al., 2011). For example, COX-2 was significantly stimulated by H/R in H9C2 cardiomyocytes by activating NF-κB and inhibition of COX-2 could attenuate H/R-induced cell apoptosis through blocking the expression of proinflammatory cytokines (Pang et al., 2016). In the ISO-induced cardiac hypertrophy rat model, COX-2 was highly expressed, accompanied by elevation of cardiac NF-κB, TNF-α, and IL-6 (Liu et al., 2012). Similarly, COX-2 expression was higher in thrombin-induced hypertrophy in primary human cardiomyocytes (Chien et al., 2015). In this study, the PPI network also showed that COX-2 was associated with the NF-κB pathway and IL-8, indicating that COX-2 could induce persistent inflammation, which plays a pathogenic role in chronic heart failure by influencing heart contractility, inducing hypertrophy and promoting apoptosis, contributing to myocardial remodeling.

In this study, bioinformatics analysis showed that COX-2 was up-regulated in the blood sample of patients with non-ischemic heart failure, indicating it might be as a promising candidate for the diagnosis of HF. Our results showed the level of COX-2 was significantly up-regulated in the non-ischemic heart failure. Plasma COX-2 also displayed potent predictive power for non-ischemic heart failure, but its sensitivity and specificity were not high enough. Micro RNAs are also present in the circulation and stable, those properties make them be potential biomarkers. In this study, we predicted that miR-4649-3p and miR-1297 could target at COX-2 by using omics bean analysis which combined with the results from 5 databases; TargetScan, PicTar, RNA22, PITA, and miRanda. It has been found that miR-1297 suppressed colorectal cancer growth by targeting COX-2 *in vitro* and *in vivo* study (Chen et al., 2014). In this study, miR-1297 was decreased in the plasma of patients with non-ischemic heart failure and negatively correlated with COX-2. Although there was a week correlation of miR-1297 and COX-2 in the plasma, miR-1297 displayed higher predictive power than COX-2. MiR-4649 was also predicted to target COX-2 and increased in the plasma of patients with non-ischemic heart failure. There was a positive correlation of miR-4649 with COX-2 in the plasma of patients with non-ischemic heart failure, indicating that there was negative feedback regulation between miR-4649 with COX-2. Notably, the COX-2 is not constitutively present in tissues and not very stable in plasma (Mendes et al., 2012; Kirkby et al., 2013). Besides, the genes or micro RNAs in tissues and blood are not always consistent (An et al., 2019). Those might lead to the week correlation of miR-4649 with COX-2 in plasma of non-ischemic heart failure. However, miR-4649 displayed stronger predictive power than COX-2 and miR-1297. Micro RNAs are also present in the circulation and stable, indicating that plasma miR-4649 and miR-1297 might be as biomarkers for non-ischemic heart failure.

Nonsteroidal anti-inflammatory drugs (NSAIDs), which inhibit the expression of COX-2, have been used to treat heart failure, but impair renal function (Bleumink et al., 2003). COX-2 selective inhibitors have similar effects on renal function as the traditional NSAIDs, and can likewise be expected to increase the risk of heart failure in susceptible patients (Patrono, 2016). The micro RNAs target COX-2 might be also used to treat heart failure. It was favored that therapeutic cardiac-targeted delivery of miR-1 reverses pressure overload-induced cardiac hypertrophy and attenuates pathological remodeling. Therefore, manipulation of miR-4649 or miR-1297 might be a strategy to cure the non-ischemic heart failure by targeting COX-2.

## Conclusion

The COX-2 related micro RNAs miR-4649, miR-1297 could be used as biomarkers for non-ischemic heart failure.

### Limitations

In this study, we used the mining strategy to identify the COX-2 and it micro RNAs, which might be used as biomarkers for non-ischemic heart failure. Although the miR-4649 and miR-1297 are predicted to target the COX-2, their correlations were week. Further studies were need to confirm that their direct correlations. In addition, the sample size was small and analyzed from a single-center, larger studies are needed to confirm the current results.

## Data Availability Statement

The raw data supporting the conclusions of this article will be made available by the authors, without undue reservation, to any qualified researcher.

## Ethics Statement

The studies involving human participants were reviewed and approved by the study protocol was approved by the ethics committee of Jilin University second hospital. Written informed consent to participate in this study was provided by the participants’ legal guardian/next of kin. Written informed consent was obtained from the individual(s), and minor(s)’ legal guardian/next of kin, for the publication of any potentially identifiable images or data included in this article.

## Author Contributions

JW designed the study. YY analyzed the data for GEO data sets and drafted the manuscript. DS collected the samples and tested the plasma levels of COX-2. XZ tested the micro RNA levels. GH performed the statistical analyses.

## Funding

This study was supported by the National Natural Science Foundation of China (31600728), Key Scientific and Technological Project of Science and Technology Department of Jilin Province (20170402032YY) and Special Fund Project of Jilin Provincial Innovation (2017C049).

## Conflict of Interest

The authors declare that the research was conducted in the absence of any commercial or financial relationships that could be construed as a potential conflict of interest.

## References

[B1] An T. Fan T. Zhang X. Q. Liu Y. F. Huang J. Liang C. (2019). Comparison of Alterations in miRNA Expression in Matched Tissue and Blood Samples during Spinal Cord Glioma Progression. Sci. Rep. 9 (1), 9169. 10.1038/s41598-019-42364-x 31235820 PMC6591379

[B2] Barrett T. Suzek T. O. Troup D. B. Wilhite S. E. Ngau W. C. Ledoux P. (2005). NCBI GEO: mining millions of expression profiles—database and tools. Nucleic Acids Res. 33 (suppl_1), D562–D566. 10.1093/nar/gki022 15608262 PMC539976

[B3] Bleumink G. S. Feenstra J. Sturkenboom M. C. Stricker B. H. C. (2003). Nonsteroidal anti-inflammatory drugs and heart failure. Drugs 63 (6), 525–534. 10.2165/00003495-200363060-00001 12656651

[B4] Camacho M. Rodríguez C. Guadall A. Alcolea S. Orriols M. Escudero J. R. (2011). Hypoxia upregulates PGI-synthase and increases PGI2 release in human vascular cells exposed to inflammatory stimuli. J. Lipid Res. 52 (4), 720–731. 10.1194/jlr.M011007 21296955 PMC3284164

[B5] Chen P. Wang B. L. Pan B. S. Guo W. (2014). MiR-1297 regulates the growth, migration and invasion of colorectal cancer cells by targeting cyclo-oxygenase-2. Asian Pac J. Cancer Prev. 15 (21), 9185–9190. 10.7314/APJCP.2014.15.21.9185 25422199

[B6] Chien P. T. Y. Lin C. C. Hsiao L. D. Yang C. M. (2015). Induction of HO-1 by carbon monoxide releasing molecule-2 attenuates thrombin-induced COX-2 expression and hypertrophy in primary human cardiomyocytes. Toxicol. Appl. Pharmacol. 289 (2), 349–359. 10.1016/j.taap.2015.09.009 26385185

[B7] Dai Z. Aoki T. Fukumoto Y. Shimokawa H. (2012). Coronary perivascular fibrosis is associated with impairment of coronary blood flow in patients with non-ischemic heart failure. J. Cardiol. 60 (5), 416–421. 10.1016/j.jjcc.2012.06.009 22867802

[B8] Dickinson B. A. Semus H. M. Montgomery R. L. Stack C. Latimer P. A. Lewton S. M. (2013). Plasma microRNAs serve as biomarkers of therapeutic efficacy and disease progression in hypertension-induced heart failure. Eur. J. Heart Failure 15 (6), 650–659. 10.1093/eurjhf/hft018 23388090

[B9] Endo K. Naito Y. Ji X. Nakanishi M. Noguchi T. Goto Y. (2013). MicroRNA 210 as a biomarker for congestive heart failure. Biol. Pharm. Bull. 36 (1), 48–54. 10.1248/bpb.b12-00578 23302636

[B10] Gathright E. C. Goldstein C. M. Josephson R. A. Hughes J. W. (2017). Depression increases the risk of mortality in patients with heart failure: a meta-analysis. J. Psychosomatic Res. 94, 82–89. 10.1016/j.jpsychores.2017.01.010 PMC5370194 28183407

[B11] Goetze J. P. Rehfeld J. F. Videbaek R. Friis-Hansen L. Kastrup J. (2005). B-type natriuretic peptide and its precursor in cardiac venous blood from failing hearts. Eur. J. Heart Failure 7 (1), 69–74. 10.1016/j.ejheart.2004.04.012 15642534

[B12] Hunt S. A. Abraham W. T. Chin M. H. Feldman A. M. Francis G. S. Ganiats T. G. (2009). 2009 Focused update incorporated into the ACC/AHA 2005 Guidelines for the Diagnosis and Management of Heart Failure in Adults A Report of the American College of Cardiology Foundation/American Heart Association Task Force on Practice Guidelines Developed in Collaboration With the International Society for Heart and Lung Transplantation. Circulation 119 (14), e391–e479. 10.1161/CIRCULATIONAHA.109.192065 19324966

[B13] Inamdar A. A. Inamdar A. C. (2016). Heart failure: diagnosis, management and utilization. J. Clin. Med. 5 (7), 62. 10.3390/jcm5070062 PMC4961993 27367736

[B14] Kirkby N. S. Lundberg M. H. Harrington L. S. Leadbeater P. D. Milne G. L. Potter C. M. (2013). Cyclooxygenase-1, not cyclooxygenase-2, is responsible for physiological production of prostacyclin in the cardiovascular system. Proc. Natl. Acad. Sci. U. S. A. 109 (43), 17597–17602. 10.1073/pnas.1209192109 PMC3491520 23045674

[B15] Linsenmayer C. Hadi A. Williams G. Doyle M. Callihan M. (2019). Polypharmacy Increases Heart Failure (HF) Readmission Rate. J. Cardiac Failure 25 (8), S26. 10.1016/j.cardfail.2019.07.072

[B16] Liu Q. Chen Y. Auger-Messier M. Molkentin J. D. (2012). Interaction between NFκB and NFAT coordinates cardiac hypertrophy and pathological remodeling. Circ. Res. 110 (8), 1077–1086. 10.1161/CIRCRESAHA.111.260729 22403241 PMC3341669

[B17] Liu M. Chen J. Huang D. Ke J. Wu W. (2014). A meta-analysis of proinflammatory cytokines in chronic heart failure. Heart Asia 6 (1), 130–136. 10.1136/heartasia-2013-010484 27326188 PMC4832715

[B18] Liu S. Yao X. Zhang D. Sheng J. Wen X. Wang Q. (2018). Analysis of transcription factor-related regulatory networks based on bioinformatics analysis and validation in hepatocellular carcinoma. BioMed. Res. Int. 2018, 1431396. 10.1155/2018/1431396 30228980 PMC6136478

[B19] Maegdefessel L. (2014). The emerging role of micro RNA s in cardiovascular disease. J. Internal Med. 276 (6), 633–644. 10.1111/joim.12298 25160930

[B20] Mendes R. T. Stanczyk C. P. Sordi R. Otuki M. F. dos Santos F. A. Fernandes D. (2012). Selective inhibition of cyclooxygenase-2: risks and benefits. Rev. Bras Reumatol. 52 (5), 767–782. 10.1590/S0482-50042012000500011 23090376

[B21] Pang L. Cai Y. Tang E. H. C. Yan D. Kosuru R. Li H. (2016). Cox-2 inhibition protects against hypoxia/reoxygenation-induced cardiomyocyte apoptosis via Akt-dependent enhancement of iNOS expression. Oxid. Med. Cell. Longevity 2016, 3453059. 10.1155/2016/3453059 PMC5067333 27795807

[B22] Patrono C. (2016). Cardiovascular effects of cyclooxygenase-2 inhibitors: a mechanistic and clinical perspective. Br. J. Clin. Pharmacol. 82 (4), 957–964. 10.1111/bcp.13048 27317138 PMC5137820

[B23] Sakharkar M. K. Singh S. K. K. Rajamanickam K. Essa M. M. Yang J. Chidambaram S. B. (2019). A systems biology approach towards the identification of candidate therapeutic genes and potential biomarkers for Parkinson’s disease. PLoS One 14 (9), e0220995. 10.1371/journal.pone.0220995 PMC6728017 31487305

[B24] Senthong V. Kirsop J. L. Tang W. W. (2017). Clinical phenotyping of heart failure with biomarkers: current and future perspectives. Curr. Heart Failure Rep. 14 (2), 106–116. 10.1007/s11897-017-0321-4 PMC5357587 28205040

[B25] Weinberg E. O. Shimpo M. De Keulenaer G. W. MacGillivray C. Tominaga S. II Solomon S. D. (2002). Expression and regulation of ST2, an interleukin-1 receptor family member, in cardiomyocytes and myocardial infarction. Circulation 106 (23), 2961–2966. 10.1161/01.CIR.0000038705.69871.D9 12460879 PMC1460012

